# Common Internal Allosteric Network Links Anesthetic Binding Sites in a Pentameric Ligand-Gated Ion Channel

**DOI:** 10.1371/journal.pone.0158795

**Published:** 2016-07-12

**Authors:** Thomas T. Joseph, Joshua S. Mincer

**Affiliations:** 1 Department of Anesthesiology, Icahn School of Medicine at Mount Sinai, New York, New York, United States of America; 2 James J. Peters Veterans Affairs Medical Center, Bronx, New York, United States of America; Coll. Medicine, UNITED STATES

## Abstract

General anesthetics bind reversibly to ion channels, modifying their global conformational distributions, but the underlying atomic mechanisms are not completely known. We examine this issue by way of the model protein *Gloeobacter violaceous* ligand-gated ion channel (GLIC) using computational molecular dynamics, with a coarse-grained model to enhance sampling. We find that in flooding simulations, both propofol and a generic particle localize to the crystallographic transmembrane anesthetic binding region, and that propofol also localizes to an extracellular region shared with the crystallographic ketamine binding site. Subsequent simulations to probe these binding modes in greater detail demonstrate that ligand binding induces structural asymmetry in GLIC. Consequently, we employ residue interaction correlation analysis to describe the internal allosteric network underlying the coupling of ligand and distant effector sites necessary for conformational change. Overall, the results suggest that the same allosteric network may underlie the actions of various anesthetics, regardless of binding site.

## Introduction

Despite their well-established safety profile and extremely wide use, the mechanism of action of general anesthetics is incompletely understood, from the molecular to the whole-brain level. The study of general anesthesia is multiscale; elucidation of the molecular action of anesthetic ligands is complementary to delineation of the neural correlates responsible for their ultimate effect, i.e. loss of consciousness [1,2]. Specifically of interest in the present work is the modulation of ion channel receptor function due to reversible binding of anesthetic molecules. While anesthetic drugs likely interact with a variety of ion channel receptors, the general consensus is that the primary target in the human central nervous system is the GABA_A_ receptor, a member of the of the Cys-loop receptor superfamily that also includes glycine, nicotinic acetylcholine, and serotonin receptors. These receptors and other structurally similar ion channels are also referred to as pentameric ligand-gated ion channels (pLGICs) in that they are composed of five homologous subunits assembled around a central channel. Each subunit has both an extracellular and transmembrane domain, the latter consisting of four helices (referred to as TM1-TM4).

Pentameric ligand-gated ion channels have been shown generally to be sensitive to anesthetics. By modifying the conformational distribution of the target ion channel, anesthetic ligands can modulate ion flux through the channel, ultimately resulting in modulation of synaptic transmission and central nervous system function [1–3]. Particular conformational changes implicated include a shift in the TM2 transmembrane helices lining the pore lumen [4] and rearrangement of the extracellular domain and its interface with the transmembrane domain [5].

*Gloeobacter violaceous* ligand-gated ion channel (GLIC) is a pLGIC and bacterial homologue of the GABA_A_ receptor that has been extensively studied as a model for anesthetic function. Anesthetic binding sites on GLIC have been determined through x-ray crystallography [6,7]. Binding-related inhibition of GLIC is related to an allosteric mechanism that causes tilting of the TM2 alpha helices that line the pore of the channel, dehydrating the pore and rendering the lumen energetically hostile to ion passage [8,9], as well as interfering with a blooming motion of the extracellular domain leaflets that presumably increases the ability of the channel to admit ions [4,10]. (Propofol may also directly occlude the GLIC ion pore [11].)

Importantly, the structural elements involved in these conformational redistributions are often distant from the anesthetic binding sites, suggesting that there exists a set of protein residues in GLIC whose motions are coupled in such a way as to allow the systematic transmission of information from the anesthetic binding site to regions remote from it, as in other proteins [12]. This is termed an *internal allosteric network* and provides insight into the mechanical aspects of protein function. Perturbing one of the residues included in this network is more likely to result in a structure-wide perturbation than perturbing a residue not included in the network. Prior theoretical efforts to describe the linkages between binding and effector sites in GLIC use, respectively, a probabilistic model derived from atomic contacts [13] and a Gaussian network model [14]. These studies provide insight into this question but describe narrow pathways rather than the overall flow of information in the protein. Such networks may also be identified from an evolutionary standpoint by sequence analysis [15] but neither this nor the former studies have a detailed energetic basis. In this study we improve on these critical aspects by using an energetics-based correlational analysis used previously for PDZ2 [12], rhodopsin [16], and Argonaute [17].

Using a computational model of GLIC derived from crystal structure, we investigate the effects on GLIC conformation of binding of propofol, the major intravenous general anesthetic used in practice today. Since GLIC can also bind other structurally distinct anesthetic ligands, we generalize this result to a single-particle nonpolar ligand, which allows abstracting away the structural nuances of propofol. We demonstrate that this binding induces asymmetry in the GLIC structure and link these changes to a common internal allosteric network. Since large molecules like GLIC may have long-period vibrational modes, we employ the MARTINI coarse-grained force field [18] to enhance sampling. The energy surface is simplified and much longer timescales can be sampled. Thus, we present results on multi-microsecond-scale simulations rather than the hundreds-of-nanoseconds-scale typical of all-atom simulations. Since MARTINI provides a 4:1 mapping between atoms and coarse-grained particles, the effective simulation time likely represents roughly a 4x improvement in phase space sampling over the actual number of timesteps. This extended conformational sampling enhances the significance of our results.

## Results

### Propofol and generic nonpolar particle preferentially localize to known anesthetic binding regions in flooding simulations

We first investigated the ability of the coarse-grained model to admit propofol into binding sites, hypothesizing that it would localize to the crystallographic transmembrane propofol-GLIC binding site [6]. Molecular dynamics (MD) simulations were run of GLIC embedded in a lipid bilayer with 32 coarse-grained propofol molecules randomly placed in the water solvent. In this “flooding” simulation, the propofol molecules were allowed to freely diffuse for 2 μs of simulation time (S1 Movie).

As expected for hydrophobic molecules in a hydrophilic solvent at the high concentration used here, roughly half of the propofol molecules aggregated into a globule in the aqueous phase surrounding the lipid bilayer. Others readily diffused into the hydrophobic lipid bilayer without aggregation, and these tended to embed themselves near the crystallographic propofol binding site. The formation of the propofol globules occupied the majority of propofol molecules, limiting the effective concentration of propofol available to diffuse from the aqueous layer into the lipid bilayer.

**Fig 1:**
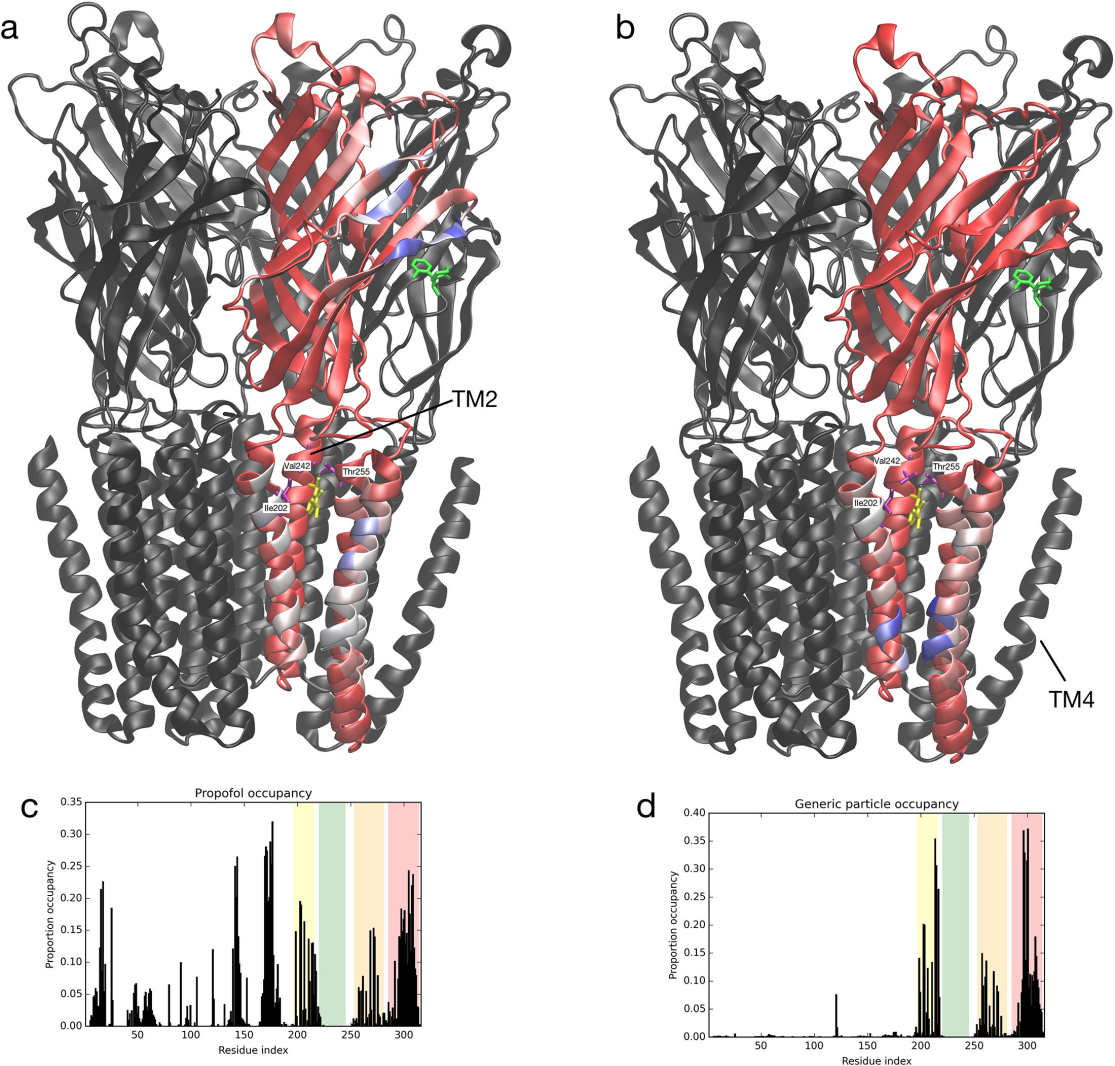
GLIC with one of five subunits colored by ligand occupancy. Red denotes lowest occupancy and blue denotes highest occupancy. Propofol occupancy is shown in (a) and generic nonpolar particle occupancy is shown in (b). For comparison, propofol (PDB 3P50) and ketamine (PDB 4F8H) are shown in their crystallographic positions determined by structural alignment of protein backbones, in yellow and green respectively. Ile202, Val242, Thr255, important in propofol binding, are indicated. Histograms of occupancy by residue index are shown in (c), (d), with domains shaded as follows: TM1: yellow, TM2: green, TM3: orange, TM4: red. Occupancy units (y-axis) are proportions with maximum 1. Occupancies are combined across subunits. Note broadly similar profiles from residue index 200 and greater, indicating an occupancy pattern in the transmembrane domain shared between propofol and the generic nonpolar particle.

**Fig 2:**
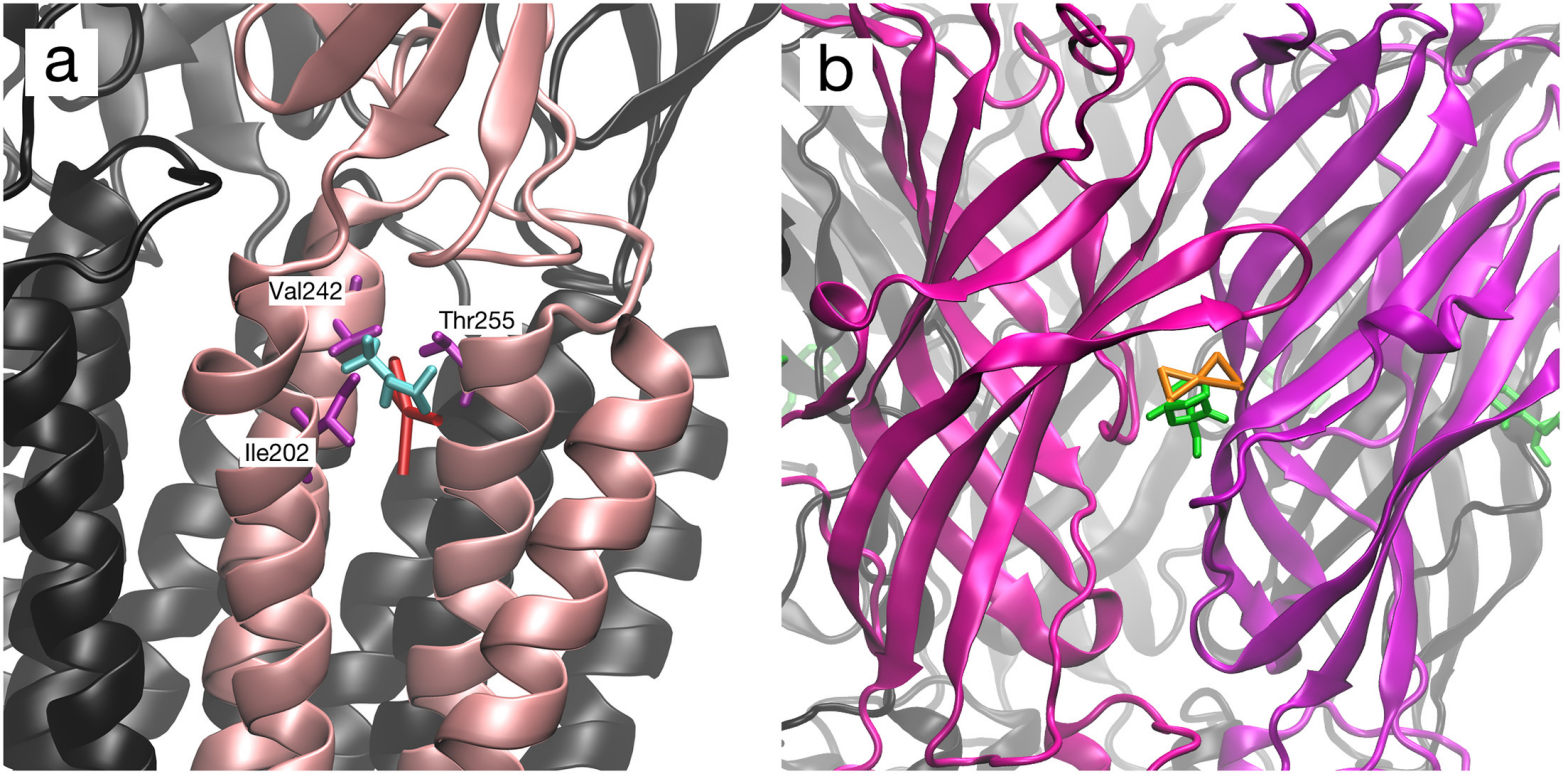
Locations of propofol in complex with GLIC. (a) Transmembrane binding site of GLIC with desflurane (blue, PDB 3P4W), with overlaid propofol from single-ligand simulation (yellow). Ile202, Val242, Thr255, important in propofol binding, are indicated. (b) Extracellular binding site of GLIC with ketamine (green, PDB 4F8H) with overlaid simulated propofol from flooding simulation (yellow). Structural alignments in both cases done by minimizing the distance between protein backbones of the crystal structure with a representative snapshot from simulation trajectory.

To more quantitatively map the localization of propofol on GLIC, we calculated the *occupancy* of propofol with respect to each GLIC residue by counting the proportion of MD trajectory snapshots during which any propofol molecule was within 7 Ångstroms of the central backbone particle of that residue. This result is depicted as a histogram in Fig 1c; since the five GLIC subunits are identical, the results are combined across subunits. In Fig 1a, the binding region is shown for one of the five identical subunits, with each residue colored by its relative occupancy. Broadly, the occupancy of those residues in and near the crystallographic transmembrane binding site was enhanced, consistent with experimental and theoretical evidence that propofol binds in this site. This selectivity is significant, especially given the simplified energy surface of the coarse-grained model. The TM4 helix, further from the binding site but with substantial interaction with the lipid bilayer, has broadly high occupancy, but no propofols were found directly adjacent to the TM2 helix lining the pore interior. In Figs 1 and 2, Ile202, Val242, and Thr255 are identified, because Ile202 and Thr255 contacts propofol in the crystal structure, and mutations in Val242 and Thr255 were shown to modify the inhibition of GLIC by propofol, even though Val242 is not in direct contact with propofol [6]. Also, in Fig 1, as a reference, Tyr23, Asn152, Phe174, are Lys183 are identified, because these are among the residues involved in the ketamine binding pocket in the crystal structure [7]—for visual clarity, Asp153, Asp154, and Leu176 are omitted.

Strikingly, high propofol occupancy was noted in the crystallographic ketamine binding site located in the extracellular domain of GLIC [7], with a single propofol continuously in that location for approximately one μs—see Fig 1A, where the crystallographic ketamine is shown for comparison. This binding pocket is amphipathic and electrostatic interactions stabilize ketamine in this site. Here, with the MARTINI force field, short-range potentials stabilized the more hydrophobic propofol in the site. Propofol localization at the ketamine binding site in our simulations generates the hypothesis that propofol might also bind GLIC in this site. Regardless, the presence of a ligand in the site does induce conformational change in GLIC (described below).

In light of the existence of a variety of hydrophobic small molecules with general anesthetic activity, we hypothesized that a simpler molecule might exhibit similar localization behavior to propofol and allow generalization of the model to remove the effects of structural nuances of propofol. A single MARTINI C4 particle, spherical and nonpolar by construction, was chosen as a generic model for a hydrophobic small molecule. We repeated the flooding simulation, using 50 generic nonpolar particles rather than propofol molecules, initially distributed randomly among the water molecules (S2 Movie). Like propofol, these particles preferentially occupied the region of the transmembrane general anesthetic binding site (see Fig 1b and 1d). As with propofol, the TM4 helix, which has substantial interaction with the lipid bilayer, had broadly high occupancy, and no generic nonpolar particles were found directly adjacent to the TM2 helix. Localization of the generic nonpolar particle to the transmembrane anesthetic binding site is consistent with the experimentally observed minimal selectivity of this site [6,7].

### Deeper transmembrane propofol binding site is recapitulated

Prior all-atom MD simulations had revealed significant mobility of propofol in the intrasubunit crystallographic cavity [6], suggesting that the crystallographic propofol binding site may not correspond to the true binding site. Subsequent all-atom simulations had shown that propofol placed in its crystallographic binding site can settle into a deeper position, closer to the TM2 helix and pore lumen [8]. This position is relatively similar to the crystallographic position of desflurane in GLIC [6].

That finding was recapitulated in our simulations. During flooding simulations, the propofol molecule was highly mobile in the crystallographic binding site and over longer timescales diffused away. Next, we wished to better explore the conformational space of the bound state. To do so, we placed one propofol bound in the crystallographic binding site, initially restrained there. After 1.2 μs of simulation time, the propofol molecule spontaneously migrated, embedding itself in the crystallographic desflurane position. A representative snapshot is shown in Fig 2a. At that point, we removed the restraints and continued the simulation. The propofol remained in the desflurane site without restraints for approximately 480 nanoseconds of production simulation time before diffusing away.

### Both propofol and generic nonpolar particle induce asymmetry in GLIC conformation

Anesthetic binding to GLIC is expected to cause conformational disruption leading to inhibition of channel function [19]. To study this effect, we conducted four simulations: GLIC with either a) a single propofol or b) a generic nonpolar particle embedded in one of the five transmembrane propofol/desflurane binding sites, c) GLIC with a single propofol embedded in one of the five extracellular ketamine binding sites, as well as d) free GLIC.

Of note, long-range electrostatics are not treated in the MARTINI force field, and therefore ligand-dependent channel dehydration effects observed in prior all-atom simulations [8] would not be expected to be observed in the present simulations. Also, because side chains are not modeled in detail and residue protonation states are not treated in MARTINI, pH-dependent effects such as proton-dependent gating in GLIC are not modeled. Despite this, the steric effects of a bound ligand on GLIC dynamics are indeed modeled and would be expected to be observed in the form of conformational change.

As described above, a single propofol molecule placed in the intrasubunit transmembrane crystallographic site remained for 480 ns, without any applied restraints, before escaping. Similarly, a single propofol placed in the extracellular ketamine site remained for 1 μs, without applied restraints, before escaping (see Fig 2b). For the analysis detailed below, we analyzed only these (non-restrained) portions of the respective simulation trajectories. The high diffusivity of the generic nonpolar particle coupled with the flattened energy surface of the coarse-grained model kept the particle from remaining in the transmembrane site over longer timescales. Therefore, to maximize sampling of the bound state to evaluate steric effects, we used a single distance restraint to anchor the generic nonpolar particle throughout that simulation, as with propofol in a position chosen by analogy with the intrasubunit transmembrane crystallographic position of desflurane in GLIC [6].

We measured the tilt in two orthogonal directions of the five pore-lining TM2 helices and of the five extracellular leaflets over the course of the simulation. The radial tilt angle measures the tilt of the major principal axis of the helix or leaflet away from the major pore axis, and the lateral tilt angle measures the tilt, tangent to the pore lumen, of the major principal axis of the helix or leaflet. We hypothesized that bound GLIC structures would exhibit a modified distribution of these angles compared to those of free GLIC.

**Fig 3:**
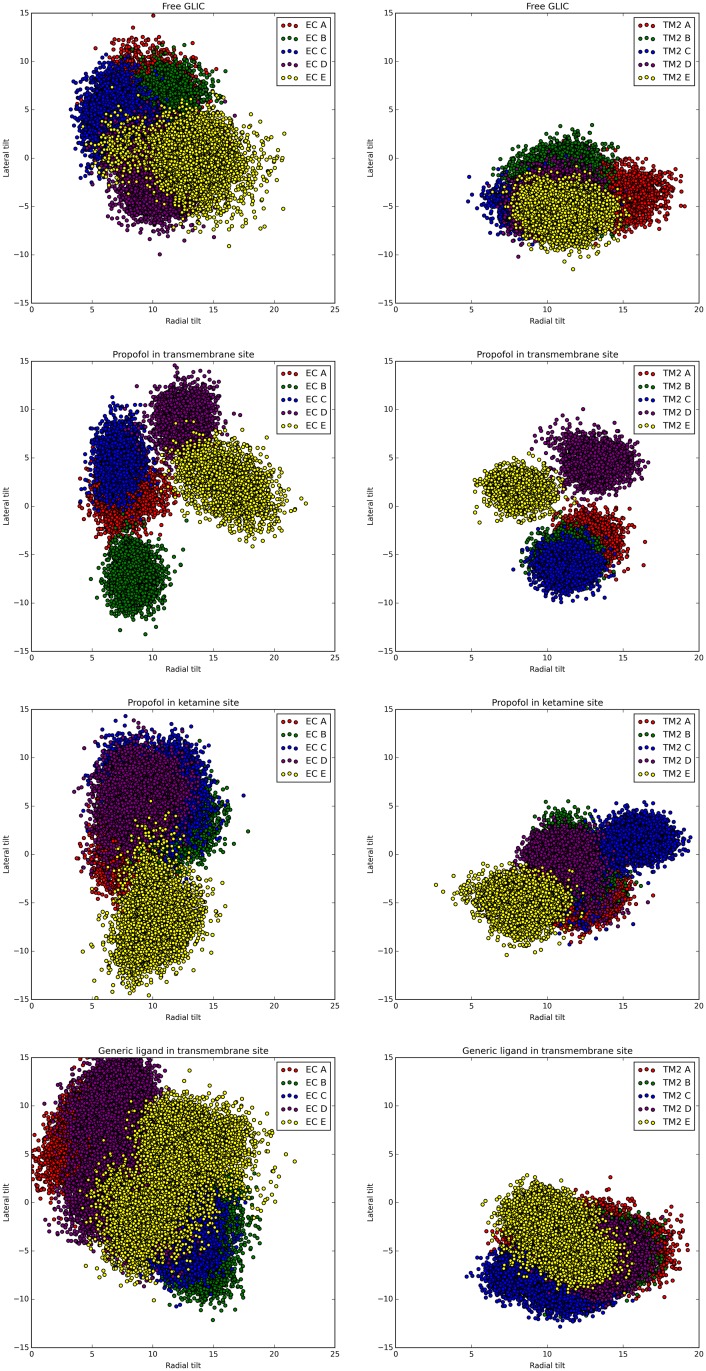
Tilt angles of key GLIC domains with and without ligand binding. Scatter plots of tilt angles of extracellular domain leaflet (left column of plots) and TM2 pore-lining helix (right column of plots) for each of the five GLIC subunits (labeled A–E), in free and bound states. The occupied transmembrane binding site is in subunit E, and the occupied extracellular ketamine binding site is between subunits E and A. Note the relatively symmetrical distribution of tilt angles in the free state and asymmetric distributions in all bound states.

In free GLIC, the TM2 helix tilt angles—plotted in Fig 3 with aggregate statistics in Table 1 –were similar across all five homomeric subunits (designated A–E), consistent with their symmetrical distribution around the central channel. When a propofol molecule was introduced into the transmembrane binding site on subunit E, TM2 helix tilts on subunits D and E, as well as most extracellular leaflet tilts, were substantially changed, inducing asymmetry. Differences in tilt angles with respect to the free GLIC structure as reported in Tables 1 and 2 are shown in Tables 3–8 for each bound GLIC structure (Δ symbol). A generic nonpolar particle in the same transmembrane site induced broad asymmetry in both extracellular and transmembrane domains. A propofol molecule in the extracellular ketamine binding site between subunits E and A also induced asymmetry in the extracellular leaflets and, significantly, in the relatively distant TM2 helices. The differences in TM2 helix and extracellular leaflet tilts between corresponding subunits in the free and each bound structure were statistically significant in all but two of 120 comparisons, using the Mann-Whitney-Wilcoxon rank sums test, with p-value threshold 0.05/120 ≈ 0.00042.

**Table 1:** TM2 helix tilt in free GLIC.

	Radial mean	Radial std. dev.	Lateral mean	Lateral std. dev.
A	13.8	1.5	-3.3	1.6
B	10.6	1.3	-1.5	1.3
C	9.8	1.4	-4.9	1.3
D	10.5	1.2	-4.2	1.4
E	11.0	1.3	-5.5	1.4

**Table 2:** Extracellular domain leaflet tilt in free GLIC.

	Radial mean	Radial std. dev.	Lateral mean	Lateral std. dev.
A	10.2	1.5	6.2	2.1
B	10.6	1.3	4.5	2.2
C	6.9	1.3	4.1	2.0
D	11.0	1.8	-1.8	2.3
E	13.4	2.2	0.2	2.1

**Table 3:** TM2 helix tilt with propofol in transmembrane site.

	Radial mean	Radial std. dev.	Lateral mean	Lateral std. dev.
A	12.9 (Δ = -1.0)	0.9	-3.3 (Δ = -0.0)	1.2
B	11.3 (Δ = 0.7)	0.9	-5.2 (Δ = -3.6)	1.1
C	11.3 (Δ = 1.4)	1.0	-6.2 (Δ = -1.3)	1.2
D	13.3 (Δ = 2.7)	1.1	4.6 (Δ = 8.7)	1.3
E	8.2 (Δ = -2.9)	1.0	1.7 (Δ = 7.2)	1.1

**Table 4:** TM2 helix tilt with propofol in ketamine site.

	Radial mean	Radial std. dev.	Lateral mean	Lateral std. dev.
A	12.5 (Δ = -1.3)	1.2	-4.1 (Δ = -0.8)	1.4
B	11.5 (Δ = 0.9)	1.0	-0.2 (Δ = 1.3)	1.6
C	15.3 (Δ = 5.5)	1.7	1.1 (Δ = 6.0)	1.8
D	11.0 (Δ = 0.5)	1.1	-0.4 (Δ = 3.8)	1.4
E	8.2 (Δ = -2.9)	1.2	-4.9 (Δ = 0.6)	1.3

**Table 5:** TM2 helix tilt with generic ligand in transmembrane site.

	Radial mean	Radial std. dev.	Lateral mean	Lateral std. dev.
A	12.3 (Δ = -1.5)	2.6	-3.9 (Δ = -0.7)	3.5
B	12.7 (Δ = 2.1)	1.8	-6.9 (Δ = -5.4)	1.8
C	10.8 (Δ = 1.0)	2.0	-8.1 (Δ = -3.2)	3.9
D	13.7 (Δ = 3.2)	1.5	-5.3 (Δ = -1.1)	3.6
E	10.3 (Δ = -0.8)	1.8	-2.9 (Δ = 2.6)	3.6

**Table 6:** Extracellular domain leaflet tilt with propofol in transmembrane site.

	Radial mean	Radial std. dev.	Lateral mean	Lateral std. dev.
A	7.7 (Δ = -2.6)	1.3	1.2 (Δ = -5.0)	1.9
B	8.4 (Δ = -2.2)	1.0	-7.3 (Δ = -11.8)	1.7
C	7.2 (Δ = 0.3)	0.9	4.9 (Δ = 0.7)	1.9
D	12.6 (Δ = 1.6)	1.2	9.0 (Δ = 10.7)	1.7
E	16.3 (Δ = 2.9)	1.9	2.3 (Δ = 2.2)	2.0

**Table 7:** Extracellular domain leaflet tilt with propofol in ketamine site.

	Radial mean	Radial std. dev.	Lateral mean	Lateral std. dev.
A	8.1 (Δ = -2.1)	1.3	1.5 (Δ = -4.6)	2.2
B	11.9 (Δ = 1.3)	1.5	4.0 (Δ = -0.5)	2.1
C	10.6 (Δ = 3.7)	2.0	7.2 (Δ = 3.1)	2.3
D	8.8 (Δ = -2.2)	1.6	5.8 (Δ = 7.5)	2.3
E	10.3 (Δ = -3.1)	1.5	-6.7 (Δ = -6.8)	2.6

**Table 8:** Extracellular domain leaflet tilt with generic ligand in transmembrane site.

	Radial mean	Radial std. dev.	Lateral mean	Lateral std. dev.
A	4.7 (Δ = -5.6)	1.3	6.1 (Δ = -0.1)	3.8
B	13.4 (Δ = 2.8)	1.5	-2.8 (Δ = -7.3)	4.5
C	11.3 (Δ = 4.4)	2.0	-1.4 (Δ = -5.5)	4.3
D	7.2 (Δ = -3.8)	1.8	6.3 (Δ = 8.1)	6.4
E	11.8 (Δ = -1.6)	2.3	4.0 (Δ = 3.9)	5.1

Overall, the presence of a ligand induces asymmetry in GLIC in both the transmembrane and extracellular domains. This is the case with all binding conditions studied, whether propofol in the transmembrane site—with magnitudes of tilt angle changes consistent with earlier simulations [8]–or in the extracellular site, or the generic nonpolar particle in the transmembrane site.

### Intrinsic internal allosteric network mediates information transfer within GLIC

As described above, the ability of ligand binding to induce conformational change at distant sites implies the existence of an internal allosteric network that mediates this transduction. To determine the pattern of energetically coupled pathways in GLIC, as well as the residues which are most significant in these pathways, we used the *interaction correlation* method [16,17], described in the Methods section. Briefly, correlations among pairs of residue interaction energies over the course of a given MD simulation were calculated and projected back onto the GLIC structure to produce a (symmetric) *residue correlation matrix R*, where each *R*_*ij*_ is a score describing the degree of correlation between residues *i* and *j*. These were calculated for each of the four binding conditions described above: free GLIC, GLIC with propofol in the transmembrane site, GLIC with generic nonpolar particle in the transmembrane site, and GLIC with propofol in the extracellular site.

**Fig 4:**
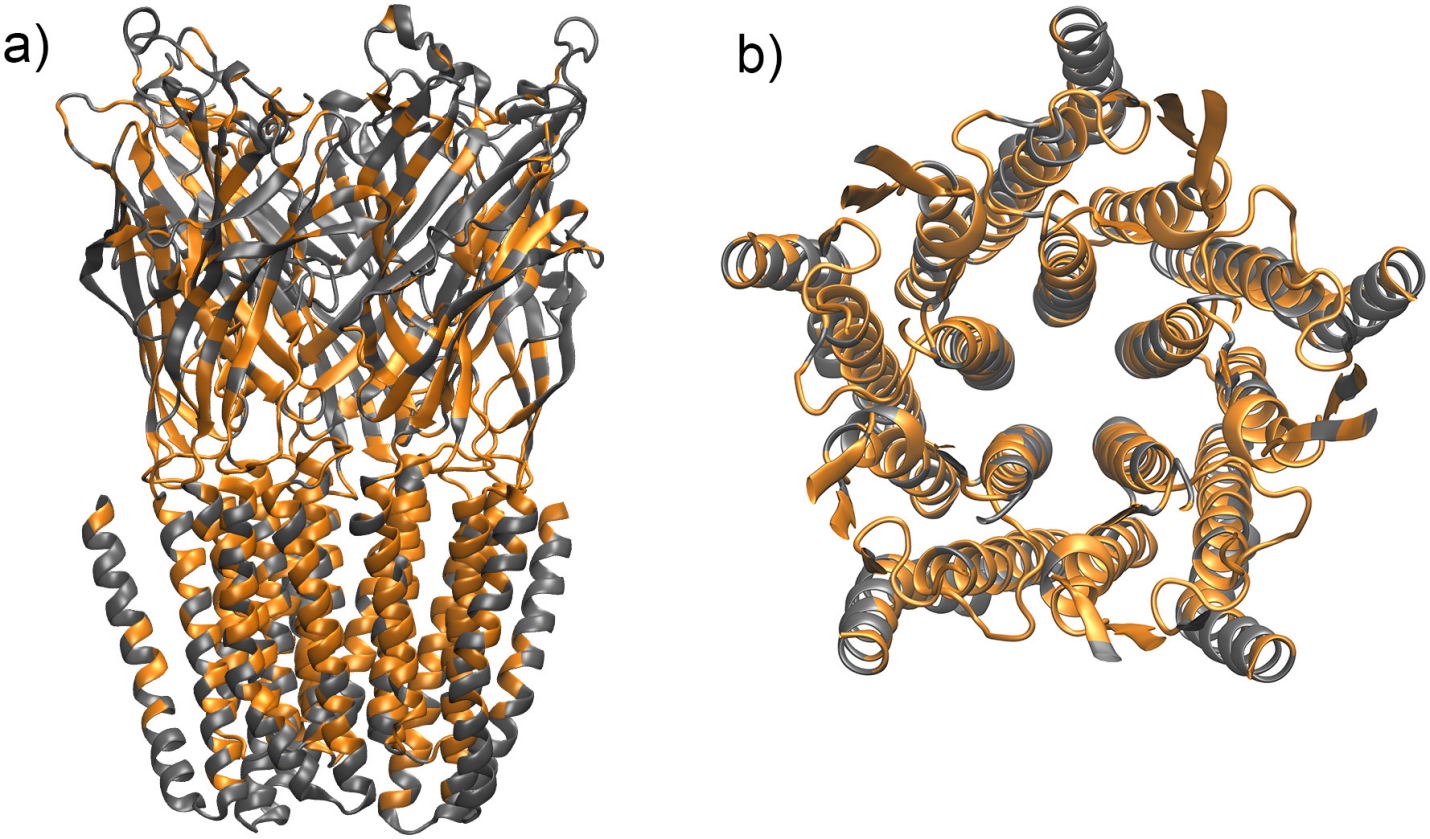
Visualizing the consensus residue correlation matrix: regions of high and low correlation scores. Residues of low correlation score are colored grey, while those of high correlation score are colored orange. Note the distribution of high-correlation residues in the transmembrane and EC-TMD interfacial regions in both (a) side view and (b) view through the pore lumen, looking in from the extracellular side.

Regardless of binding condition, there were a large number of significantly interacting residue pairs. This can be attributed to the dense organization of GLIC itself: as each residue has relatively many interacting neighbors, there are many significant interactions represented in the residue correlation matrix. Despite this, a pattern reflecting the existence of five subunits was appreciable on inspection, regardless of binding condition (see S1 Fig). In order to extract residues that were highly correlated with a large number of other residues, we computed a consensus residue correlation matrix as the element-wise RMS average of the residue correlation matrices for all binding conditions, including free GLIC; taking the sum of each row *R*_*i*_ yields the degree of correlation of residue *i* with the entire GLIC structure. As seen in Fig 4, highly correlated residues tended to be located in the extracellular-transmembrane (EC-TMD) domain interface and transmembrane domains, with a limited number of residues in the extracellular domain. Residues with relatively low correlation scores included the majority of the extracellular domain and parts of the transmembrane helices, including the pore-lining residues and those residues in contact with the lipid bilayer.

We next attempted to characterize individual allosteric pathways by clustering the residues using the correlation values as the distance metric (e.g. the distance between two residues *i* and *j* is defined *D*_*ij*_ = *R*_*ij*_) [12]. This resulted in nearly all highly correlated residues being placed into a single monolithic cluster, essentially providing no additional information. Prior application to a large, dense protein had given similar results, suggesting that standard clustering using this simple distance metric would not provide a meaningful partitioning of the structure, due to the high density of interacting residues [17]. In effect, the pattern of allostery in a dense protein such as GLIC is not easily reduced to a few linear paths connecting distant residues (though this conception may be appropriate for smaller, less dense proteins).

**Fig 5:**
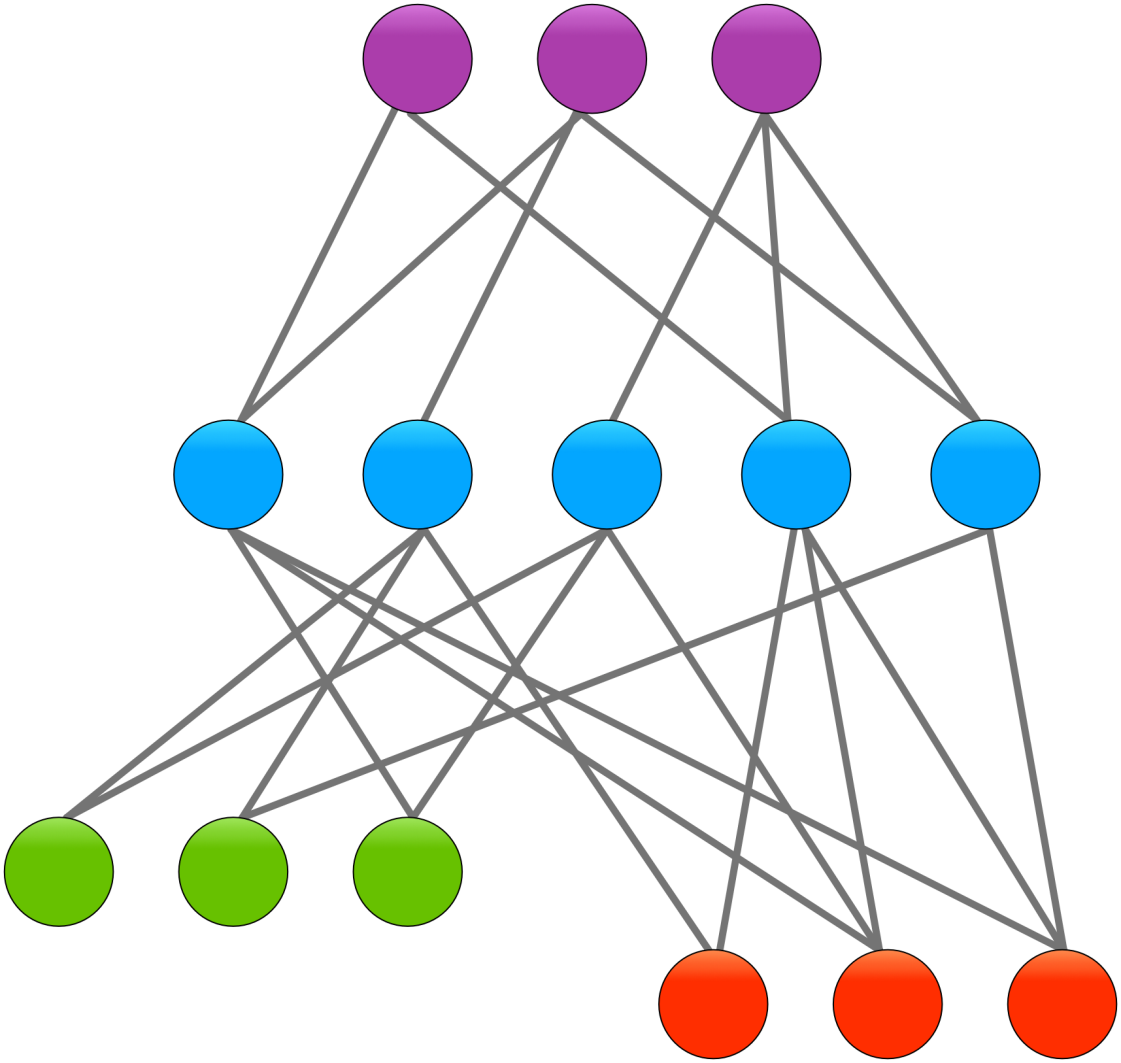
Cross-sectional clustering. Residues are segregated into different clusters based on how similar their connectivity is. Grey lines represent a high degree of correlation (*i*.*e*. high *R*_*ij*_ value). For example, all residues in the green (lower left) cluster tend to be connected to the central (blue) cluster, but not necessarily to each other. Therefore they are grouped together. But while green, red (lower right), and purple (top) clusters tend to be connected to the central cluster, their connectivities are dissimilar enough from each other that they are not grouped together.

As in the prior study of Argonaute [17], we instead regarded the residue correlation matrix as a list of arrays of observations. The *i*th row of the matrix describes the correlation of the *i*th residue with all other residues. Here we define the distance metric between two residues *i* and *j* not as a single element *R*_*ij*_ but rather as the Euclidean distance between their respective rows of observations: *D*_*ij*_ = ‖*R*_*i*_* − R*_*j*_‖. Using this metric, residues can be clustered together if they are similar in their pattern of correlation to all other residues. Rather than encompassing a specific allosteric pathway in its entirety, a cluster represents a “cross-section” across many pathways (see Fig 5). Residues in one cluster are not necessarily connected to each other, but rather show similar connectivity to residues in other clusters. This view permits a description of the overall patterns of energy/information flow within a dense structure such as GLIC where such flow does not necessarily occur along simplified linear paths.

We used divisive hierarchical clustering with this distance metric to produce clusterings of the GLIC structure for each binding condition. There were ~10 clusters located in the transmembrane and EC-TMD interfacial regions, along with various singleton clusters. Within the transmembrane and interfacial regions, we observed no clearly discernible linear mechanical pathways. Instead, the overall picture was of a highly complex allosteric network of interconnected pathways in an active “core”, surrounded by a shell of low-correlation (low connectivity) residues.

**Fig 6:**
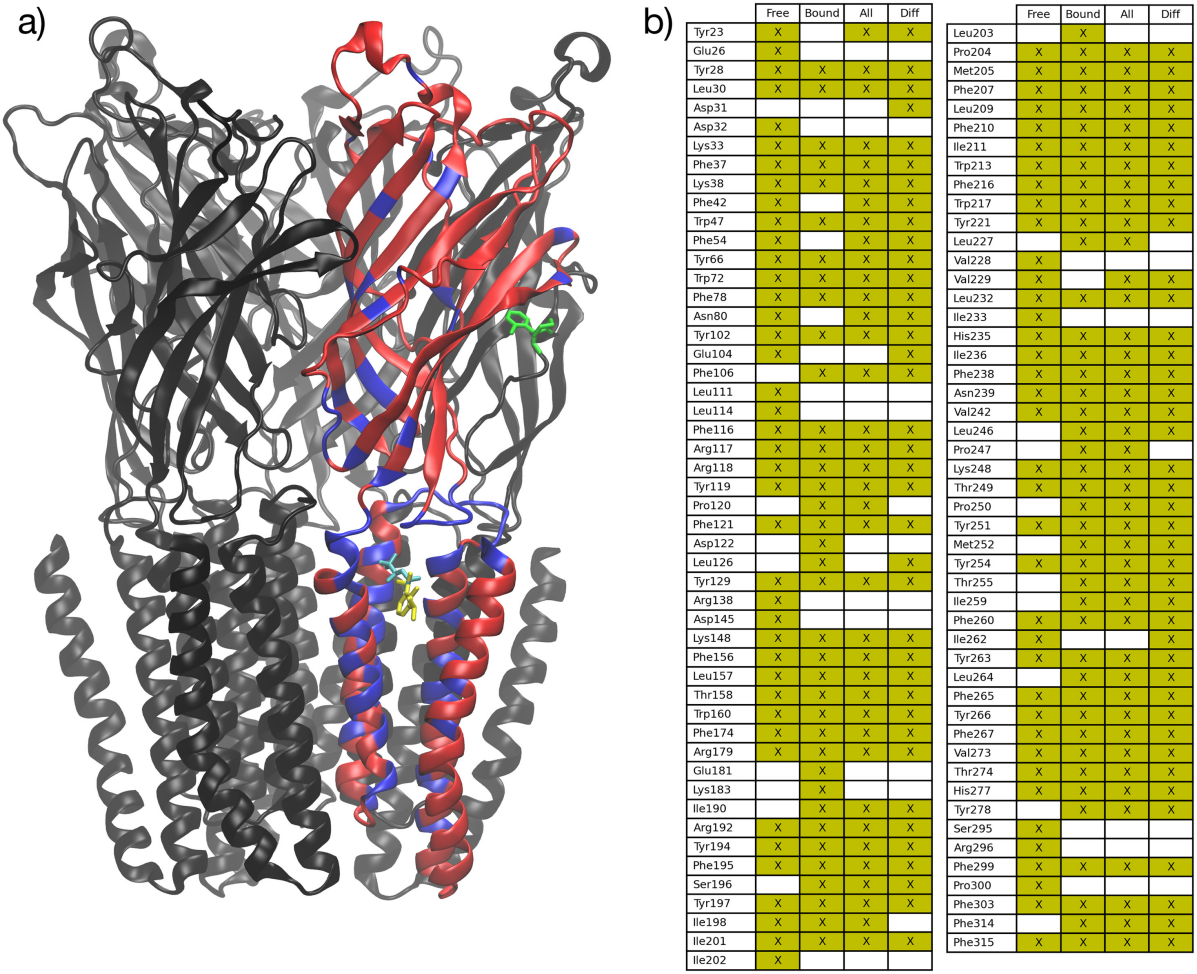
Hot-spot residues in the internal allosteric network. (a) Highly correlated residues (hot-spots) in the internal allosteric network (highlighted in blue), derived from the consensus residue correlation matrix of free GLIC and all single-ligand-GLIC simulations. (b) List of these residues, shown with an “X” mark, for various binding conditions. Note the high degree of commonality. For reference, propofol (yellow), desflurane (blue), and ketamine (green) are shown in their crystallographic binding sites.

Next, we determined which residues represented “hot-spots”: those residues with the highest degree of correlation with other residues would be expected to have the most influence on the structure if perturbed. Summing the *i*th row or column of the correlation matrix *R* (or a consensus matrix) yields a per-residue vector assigning a correlation score to each residue. The highest-scoring residues, chosen as those above the 75th percentile, are hypothesized to comprise the key residues (or hot-spots) in the internal allosteric network. These residues for free GLIC are listed in Fig 6 (column “Free”). Also listed are the highest-scoring residues derived from the RMS average of the residue correlation matrices of all bound conditions (“Bound”), free and all bound conditions (“All”), and the RMS mean of the difference between ligand-bound and free GLIC residue correlation matrices (“Diff”).

Most hot-spot residues were located within the transmembrane domain and at the EC-TMD interface. Several residues were identified in the transmembrane general anesthetic binding site; among them were Tyr197 and Ile201. Notably, Pro250 was identified, along with both its sequence-adjacent neighbors. This highly-conserved proline is in the TM2-TM3 linker and functions as a hinge in the tilting motion of the pore-lining TM2 helix [4,10]. In the EC-TMD interface, residues in the β6-β7 linker, the C-terminal region of the TM4 helix, and the pre-TM1 region were identified. These are the same regions implicated experimentally in structural rearrangement during GLIC gating [5]. In addition, the β1-β2 linker was identified, similarly implicated in pLGIC gating [4].

**Fig 7:**
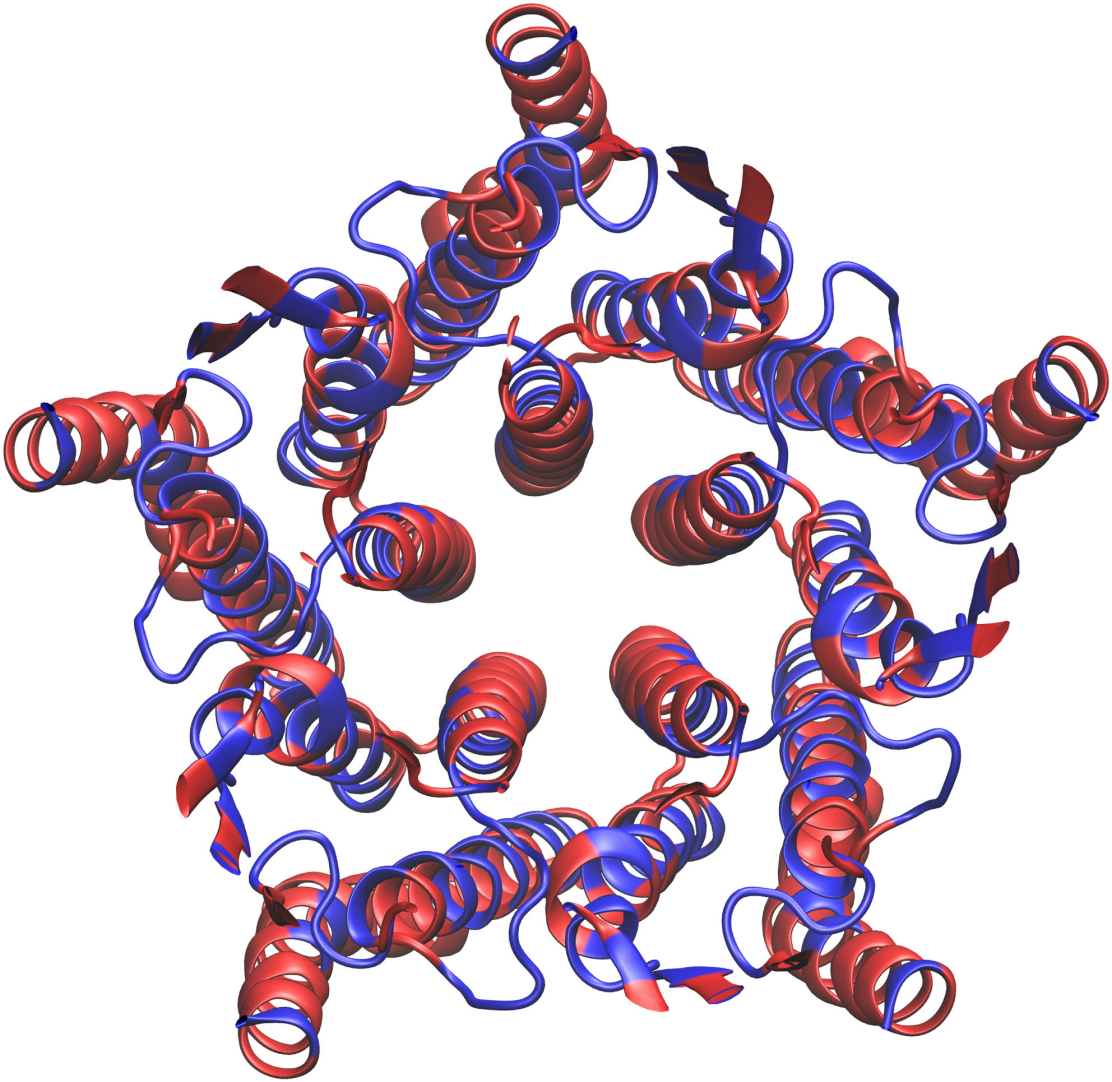
Hot-spots in the transmembrane domain. View of the transmembrane domain from the extracellular side. Highly correlated residues are blue. Note the lack of such residues lining the lumen or exposed to the lipid bilayer, describable as a tube-like geometry in which hot-spots are located within the volume of the tube, but not on either inner or outer surfaces.

Residues lining the pore lumen were not identified as network hot-spots (Fig 7), indicating that the channel is resistant to perturbations induced by ions passing through it. Additionally, the TM4 helix was largely spared on the external face with substantial exposure to the lipid membrane. However, key residues shown experimentally to be involved in TM4-TM1 and TM4-TM3 interactions in GLIC (Phe314, Phe315, Tyr254, Phe265, Tyr266) as well as Phe residues in TM4 that are conserved between ELIC and GLIC [20] are fully predicted. Within the transmembrane domain, the overall geometry that emerges is that of a hollow cylinder or tube, in which hot-spot residues are located within tube volume, but not on either its inner or outer surface.

Two residues located in the extracellular crystallographic ketamine binding site, Tyr23 and Phe174 [7], ranked highly. This, and the general similarity in results for propofol bound in either transmembrane or extracellular sites, suggests that ketamine binding may induce conformational change by utilizing the same allosteric network as propofol and the generic nonpolar ligand even though its binding site is remote from that of propofol. The results are also concordant with recent crystallographic evidence of acetate binding in the extracellular domain [21] although only Y102 and E104 are identified here.

Notably, as illustrated in Fig 6b, there is a high degree of commonality between the set of hot-spot residues for free GLIC (“Free”) and a consensus residue correlation matrix derived from the various binding conditions (“Bound”). Comparing the top-ranking quartile of residues in the respective matrices, 76% (60 of 79) are identical. This indicates that key elements of the network are preserved between free and bound states.

Finally, we asked a subtly different but related question: is the internal allosteric network preserved upon ligand binding? In other words, which residues undergo the greatest change in correlation upon ligand binding, and are these the same residues that have high correlation scores in both bound and free structures? To address this question, we derived a difference residue correlation matrix as the root-mean-square average of the element-wise difference between each bound-ligand residue correlation matrix and the free GLIC residue correlation matrix. The residues found by summing as above are listed in Fig 6 (column “Diff”). These are the residues which undergo the greatest change in correlation score upon ligand binding. Strikingly, 95% (75 of 79) of the top quartile of residues were shared with those derived from the total consensus matrix described above (column “All”). This suggests, intuitively, that those residues which are most highly involved in the internal allosteric network are also the ones which are most affected in ligand binding. This result is consistent with our understanding of allostery: the dynamics of key residues are modified by ligand binding, and coupling between these residues enables propagation of a dynamical change across the structure from ligand binding site to effector site.

## Discussion

There is significant crystallographic evidence that multiple general anesthetics may bind to GLIC in the same transmembrane site [6]. Our flooding simulations, similar in philosophy to prior simulations studying isoflurane binding sites [22], recapitulate the propensity of propofol to localize to the site, and predict that a small, generic nonpolar ligand could also be admitted to this site. Although the coarse-grained model has limited ability to quantitatively predict binding affinities for protein-ligand interactions [23], the propensity for ligands to localize to this site nonetheless reflects a relative energy minimum. Notably, the high-occupancy region is also congruent with general anesthetic binding sites identified by docking studies on apoferritin and GLIC [24], as well as by MD studies of isoflurane [25]. Localization of the generic nonpolar particle to this site in our simulations represents a novel prediction that is consistent with experiments showing inhibition of GLIC by xenon [26] as well as crystallographic evidence of xenon binding to hydrophobic sites in various proteins [27]. Although our generic particle is not polarizable and therefore has limited direct application as a model for xenon, this result may still be generalized to suggest surprisingly minimal selectivity for small, hydrophobic molecules in the transmembrane site.

The propensity for propofol to localize to the crystallographic ketamine binding site in the extracellular domain during the flooding simulation was interesting. In a subsequent simulation, a single propofol molecule remained embedded in the ketamine site without external restraints for a significant amount of simulation time, suggesting that it is stable there, as opposed to being purely an artifact of an artificially high propofol concentration. The ketamine binding site is analogous to (but slightly displaced from) the extracellular intersubunit orthosteric binding site observed in similar pLGICs, such as the nicotine binding site on LS-AChBP [28], the acetylcholine binding site on ELIC [29], the glutamate binding site in GluCl [30], and the benzamidine binding site on the human GABA_A_ receptor [31]. The predicted binding of propofol at this site is concordant with existing evidence of nonselective binding there. In particular, besides ketamine, GLIC can bind a variety of molecules at this site, including acetate and cinnamic acid derivatives [21,32]. Although this finding merits future study with a more accurate electrostatics model, it provides a molecular explanation for observed antagonism between ketamine and propofol [33,34]. Clinically, the antagonistic effect may be limited, however, by the relatively hydrophobic propofol being more likely than ketamine to partition into the lipid bilayer and preferentially access the transmembrane site.

Once bound, in our simulations, both propofol (in either transmembrane or extracellular site) and the generic nonpolar particle induce asymmetric changes in the orientations of the pore-lining TM2 helices and the extracellular domain leaflets. Conformational changes in these domains are critical to the gating mechanism that modulates ion flux through the channel. Specific changes previously proposed involve a quaternary twisting motion of the pore-lining helices, tilting of the extracellular domain beta sheets, and rearrangement of the EC-TMD interface. In our simulations, substantial asymmetry in these structural elements was induced by anesthetic binding, which would disrupt those conformational changes implicated in ion passage [8]. For this to happen, information must be transmitted from the binding site across the structure to the domains of GLIC whose motion has changed. The overall architecture of the allosteric network shown through our clustering analysis, based on energetic correlations between residues, is concordant with the intuitive direction of information flow implied by this mechanism. In particular, the EC-TMD interfacial region, known to be critical for conformational change, is central to the network. We described a subset of residues of primary importance in this allostery, distributed across the transmembrane domains and EC-TMD interface, but not including the majority of the extracellular domain or the pore lumen. Notably, this internal allosteric network, native to free GLIC, is preserved among the multiple binding conditions we studied, even though the ligand-bound conformations are modified.

Prior work on information transfer in GLIC that addressed the question of how information is transferred from the extracellular domain to key residues in the interfacial domain is largely probabilistic in nature and traces a path between these defined endpoints. Our method reflects a different but complementary perspective: that internal allostery is mediated in large part by the convergence of a large number of small interactions, particularly in densely packed structures such as ion channels. This line of reasoning is motivated by studies suggesting that a linear mechanical pathway may not always underlie internal allostery, which is instead governed by more complex patterns of energetic coupling [35,36]

Allosteric hot-spot residues identified in our analysis are in substantial agreement with experimental evidence implicating specific residues in structural rearrangement during GLIC gating [5] as well as in TM4 helix interactions critical to channel function [20], and highlight residues that comprise various anesthetic binding sites, whether the crystallographic transmembrane propofol/desflurane site, the crystallographic extracellular ketamine site, or EC-TMD residues found to be important in docking studies of thiopental and halothane with GLIC [37,38]. Importantly, in our simulations, propofol binding at both the transmembrane (*e*.*g*. propofol/desflurane) and extracellular (*e*.*g*. ketamine) sites, even though they are remote from each other, reveals similar allosteric architecture and hot-spot residues, including residues at both binding sites. This further suggests that anesthetic binding at both sites employs the same allosteric network to globally effect GLIC structure and dynamics. Our results thus provide a theoretical underpinning for existing experimental data and provide a link among the modes of action of various general anesthetics through a common internal allosteric network to elicit functionally important conformational change. While all anesthetics have a spectrum of interactions with various proteins, we demonstrate here a significant commonality in an important mechanistic pathway.

Recently, structures of the structurally homologous human GABA_A_ receptor have been solved, albeit only with one subunit composition to date [31]. The model we present provides a framework for evaluating the GABA_A_ receptor internal allosteric network as further structural data about other subunit compositions and anesthetic binding modes becomes available.

## Methods

### Model construction

Starting with crystal structure of GLIC bound to propofol (PDB: 3P50) [6], an all-atom model of free GLIC was constructed, embedded in a lipid bilayer placed according to the Orientations of Proteins in Membranes database [39]. Compared to a newer crystal structure of the open form of GLIC [21], the RMSD of the backbone is 0.4 Å, well within the degree of fluctuation expected during molecular dynamics simulation. Sodium and chloride ions were added for electroneutrality and an ion concentration for each of ~0.15 M. This was converted to a coarse-grained representation using the MARTINI 2.1 force field with polarizable waters and an ELNEDIN elastic network scaffold, with a force constant of 500 kJ mol^-1^ nm^-2^ to preserve secondary structure [18,40]. (Significantly lower force constants, in trial simulations, permitted the unrealistic deformation of the structure.) The coarse-grained representation of propofol, consisting of five coarse-grained sites, was the same as in a prior study [41]. The generic nonpolar ligand was represented by a single MARTINI C4 particle, chosen for its apolar parameterization and similar oil:water partition coefficient in our simulations to the accepted value for xenon (27:1 lipid:water in simulation to 20:1 accepted value), which has general anesthetic activity. Although, unlike xenon, the C4 particle is not polarizable, we wished to choose a generic particle with some similarity to a known general anesthetic. In flooding simulations, either propofol or generic nonpolar particles were initially placed randomly in the bulk water by replacement. In single-ligand simulations, ligands were initially placed by analogy with crystal structures (propofol from PDB: 3P50 and ketamine from PDB: 4F8H [7]).

### Molecular dynamics simulations

Molecular dynamics simulations were conducted using GROMACS 4.6 [42] on multiprocessor computers [43]. For each simulation, minimization, gradual heating to 300 K, and equilibration were done, initially with solute position restraints which were then released, and 2 μs of production simulation trajectory was collected. The simulation timestep was 20 fs. Periodic boundary conditions were utilized. Since the effective time sampled using MARTINI is roughly 4 times that of all-atom simulations, while the actual simulation length is 2 μs, the effective simulation length is approximately 8 μs.

### Interaction correlation analysis

The interaction correlation method, previously described in detail [16,17] was used to study internal allosteric pathways in GLIC. The method reveals energetic correlations between residues over the course of a molecular dynamics simulation. Further analysis yields a list of residues that have a high degree of energetic correlation with the whole structure (hot-spots) as well as patterns of energy transfer within the GLIC structure that provide insight into the architecture of the internal allosteric network.

For each simulation trajectory, using the MARTINI force field parameters, we computed the nonbonded pairwise residue interaction energies *E*_*ij*_ for each residue pair (*i*, *j*), excluding interactions between adjacent and therefore covalently bonded residues. In MARTINI this consists only of short-range potentials, as short-range electrostatics are encoded in those parameters and long-range electrostatics are not included, so these are omitted here as well.

The *pair correlation matrix C*, consisting of the correlation over time between each pair (*i*, *j*) and (*k*, *l*) of these pairs was then calculated:

$$C_{ij,kl}=\frac{\sum_{t}\left(E_{ij}^{t}-\bar{E_{ij}}\right)\left(E_{kl}^{t}-\bar{E_{kl}}\right)}{\sum_{t}\sqrt{{\left(E_{ij}^{t}-\bar{E_{ij}}\right)}^{2}{\left(E_{kl}^{t}-\bar{E_{kl}}\right)}^{2}}}$$ (1)

where summations are over each trajectory frame *t* and overbars denote an average over those frames. Only those pairs with average interaction energy greater than 0.02 kcal/mol (83.68 J/mol) and correlation > 0.4 were kept. This was done to eliminate very low level correlations and to limit the size of the calculation, as in the worst case of all pairs being significant this results in an O(*n*^4^) calculation, which would have not been tractable for this system. Note that by calculating correlations between the interaction energies of residue *pairs* rather than pairwise interactions of the residues themselves, higher order correlations are captured than in, for example, a simple position correlation matrix. The motivation is that two residues *i* and *l* may not be directly correlated to each other, but if (*i*, *j*) is correlated to (*k*, *l*) then there is an energetic relationship between *i* and *l* that would be ignored by examining those two residues effectively in isolation, as in a strict pairwise analysis.

As *C* does not have a direct geometric interpretation, it was then projected back onto the GLIC structure to produce the *residue correlation matrix R* as follows. The residue correlation *r*_*ij*_ of a residue pair (*i*, *j*) was defined to be the sum of all pair correlation values *C*_*m*,*n*_ for which *i* was in residue pair *m* and *j* was in residue pair *n*. The delta function enforces this constraint:

$$R_{ij}=\sum_{m}^{N}\sum_{n}^{N}\left|C_{m,n}\times \delta_{m,n}^{ij}\right|$$ (2)

**Fig 8:**
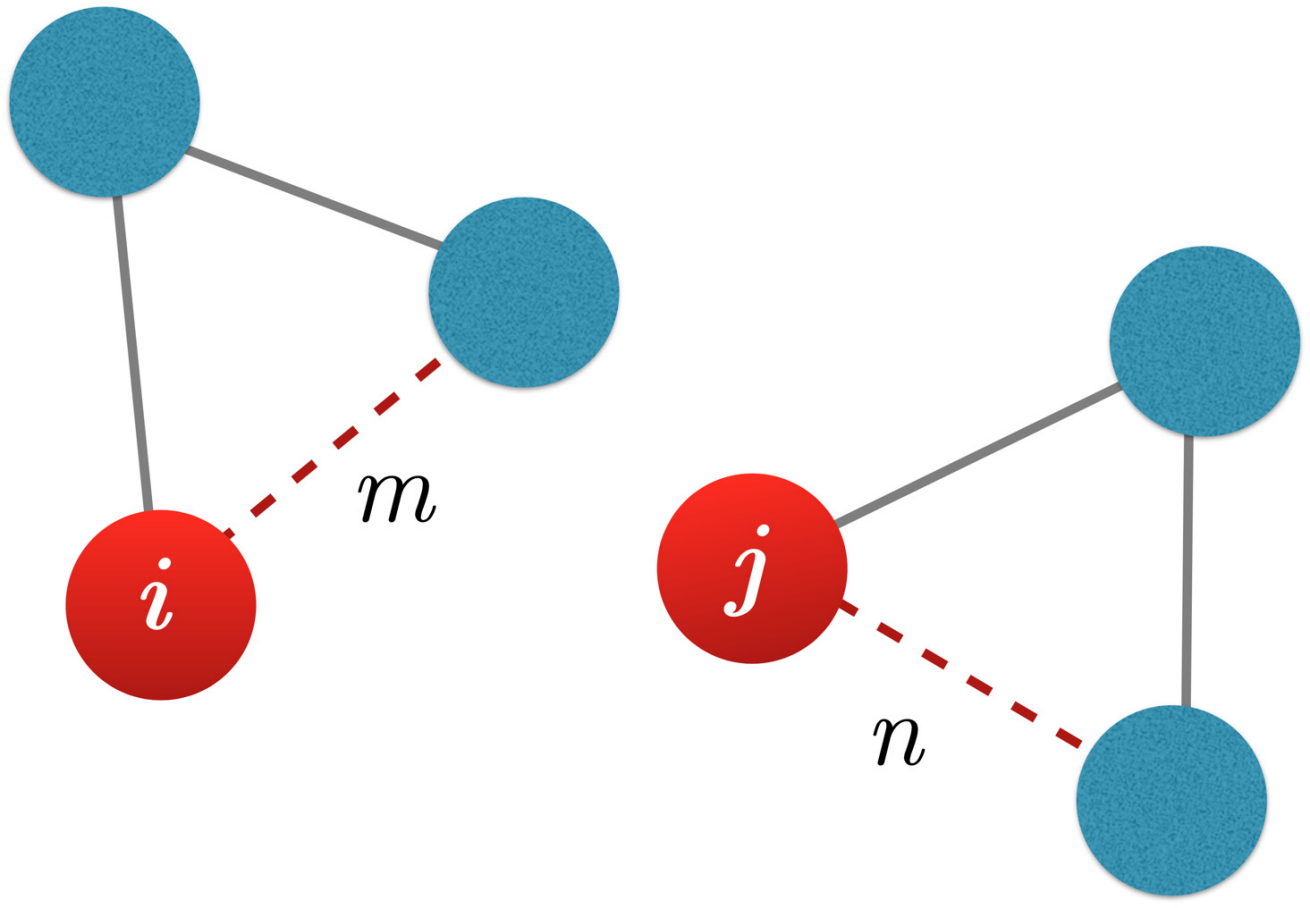
Illustration of calculation of *R*_*ij*_ with Eq 2. Two residues *i* and *j* are shown as part of a hypothetical structure. They are part of pairs *m* and *n* respectively. The degree of correlation between *i* and *j* includes the energetic correlation between *m* and *n*, even though *i* and *j* may not be otherwise closely interacting.

This is illustrated in Fig 8.

Each R_*ij*_ then describes the degree of energetic coupling between each pair of residues (*i*, *j*) in the structure (images of representative residue correlation matrices are shown in S1 Fig). Summing across any row or column in *R* yields a vector of correlation scores, one for each residue, where each score describes the degree of correlation of the corresponding residue with the rest of the structure. The residues with the highest-ranking scores are hypothesized to be hot-spots of the hypothetical internal allosteric network as sampled by the simulation. Clustering of residues based on their correlation scores (using different distance metrics based on the correlation matrix) yields information regarding the complex flow of energy within the structure and the architecture of the internal allosteric network.

## Supporting Information

S1 Fig Sample residue correlation matrices R for free GLIC and GLIC + 1 propofol. Residue indices are on each axis. Note 5x repeating pattern corresponding to the five subunits.
(TIFF)

S1 Movie Flooding molecular dynamics simulation of GLIC with 32 propofol molecules. Each frame represents 2 nanoseconds.
(MP4)

S2 Movie Flooding molecular dynamics simulation of GLIC with 50 generic particles. Each frame represents 2 nanoseconds.
(MP4)
