# Supplementary figures and images for: Common Internal Allosteric Network Links Anesthetic Binding Sites in a Pentameric Ligand-Gated Ion Channel
Source: PLoS One. 2016 Jul 12;11(7):e0158795. doi: 10.1371/journal.pone.0158795 (PMC4942068; doi:10.1371/journal.pone.0158795)

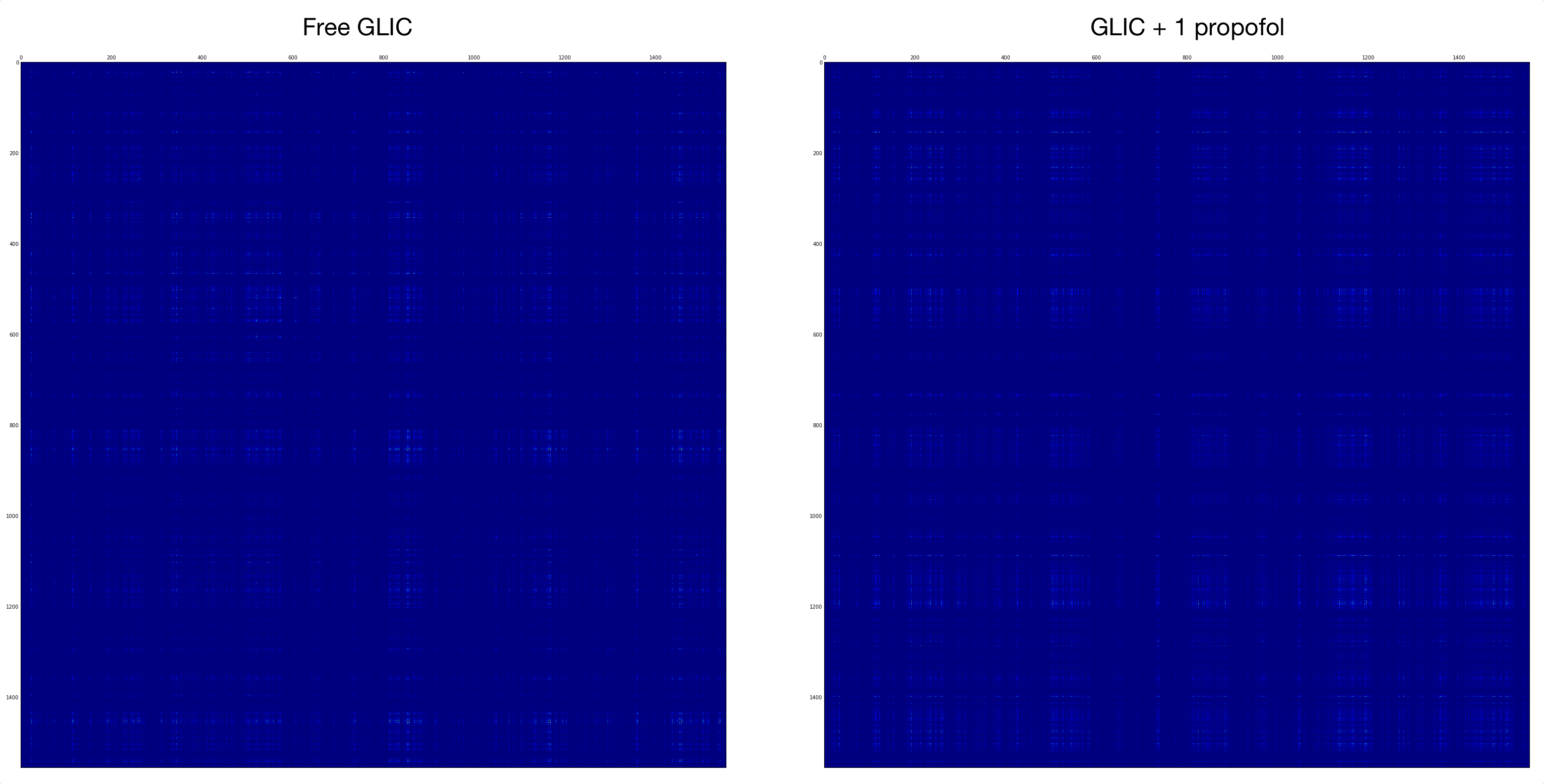

Supplement: S1 Fig — Residue indices are on each axis. Note 5x repeating pattern corresponding to the five subunits. (TIFF) [file pone.0158795.s001.tiff]
